# Psychological repercussions of PM air pollution in human aging: a comprehensive review of urban and rural environments

**DOI:** 10.3389/fphar.2025.1517090

**Published:** 2025-02-17

**Authors:** Laura O. Gallardo, Montserrat Aiger Vallés, Blanca Cativiela-Campos, Laura Domingo-Valero, Ángel Barrasa, Matilde Alique, Caridad López-Granero

**Affiliations:** ^1^ Department of Psychology and Sociology, University of Zaragoza, Teruel, Spain; ^2^ Department of Psychology and Sociology, University of Zaragoza, Zaragoza, Spain; ^3^ Departamento de Biología de Sistemas, Universidad de Alcalá, Alcalá de Henares, Madrid, Spain; ^4^ Instituto Ramón y Cajal de Investigación Sanitaria (IRYCIS), Madrid, Spain

**Keywords:** air quality, elderly, cognition, mood disorders, particulate matter, public health, rural/urban areas, social health

## Abstract

Air pollution and its effects on population health are currently among the most important public health issues. It is well established that the impact of air pollution on health is exceedingly high, although it ignores its real scope and effects on the aging process because studies on air quality have largely focused on younger age groups. Herein, we emphasize the relevance of air quality to the behavioral aging process, taking into account the place of residence - rural or urban. We raise the following question: Can air quality and residential settings modulate cognitive, emotional and social behaviors during the aging? Some studies have analyzed the role of residential settings and air pollution in the context of a behavioral frame in elderly people. Based on the analyzed literature, this revision concluded that air pollutants affect cognitive function, increasing the risk of dementia as well as depression and anxiety emotional responses. In addition, social networks and inclusion can modulate and mitigate the effects observed during the aging in rural areas that are exposed to less contamination. Although there is no consensus, it seems that some observed behavioral effects are sex-dependent, as women are more vulnerable to air pollution. Additionally, we examined why older adults are vulnerable to the health effects of Particulate Matter (PM) exposure and highlighted the importance of social health in this context. Environmental agents could be the key to understanding the susceptibility and variability observed during aging in behavioral symptoms. Although cognitive decline is related to increased age, it is not a manipulated factor. Efforts should be centered on locating factors implicated in the aging process that could be susceptible to manipulation or variation, such as the choice of the place of residence and the air that we are breathing. Given the significant societal impact of PM, research and policy regulations should be closely aligned and collaborative.

## 1 Introduction

Ambient pollution, both indoors and outdoors, and its impact on population health represent some of the most pressing environmental and public health challenges today. It is well established that the impact of air pollution on health is exceedingly high (Chen and Kan, 2008), although it ignores its real scope and effects on the aging process, since studies on air quality have largely focused on younger age groups. The World Health Organization (WHO) states that nine out of 10 people inhale air with elevated pollutant levels. Furthermore, the organization estimates that poor air quality is responsible for 7 million deaths annually (World Health Organization, 2021). In addition, household air pollution from the combustion of polluting fuel or inefficient stores also causes serious health effects, such as lung diseases, lung cancer, and cardiovascular diseases, and is believed to cause over 3 million deaths a year (WHO, 2024). Consequently, air pollution is recognized as a significant public health issue (Brugha and Grigg, 2014).

**FIGURE 1:**
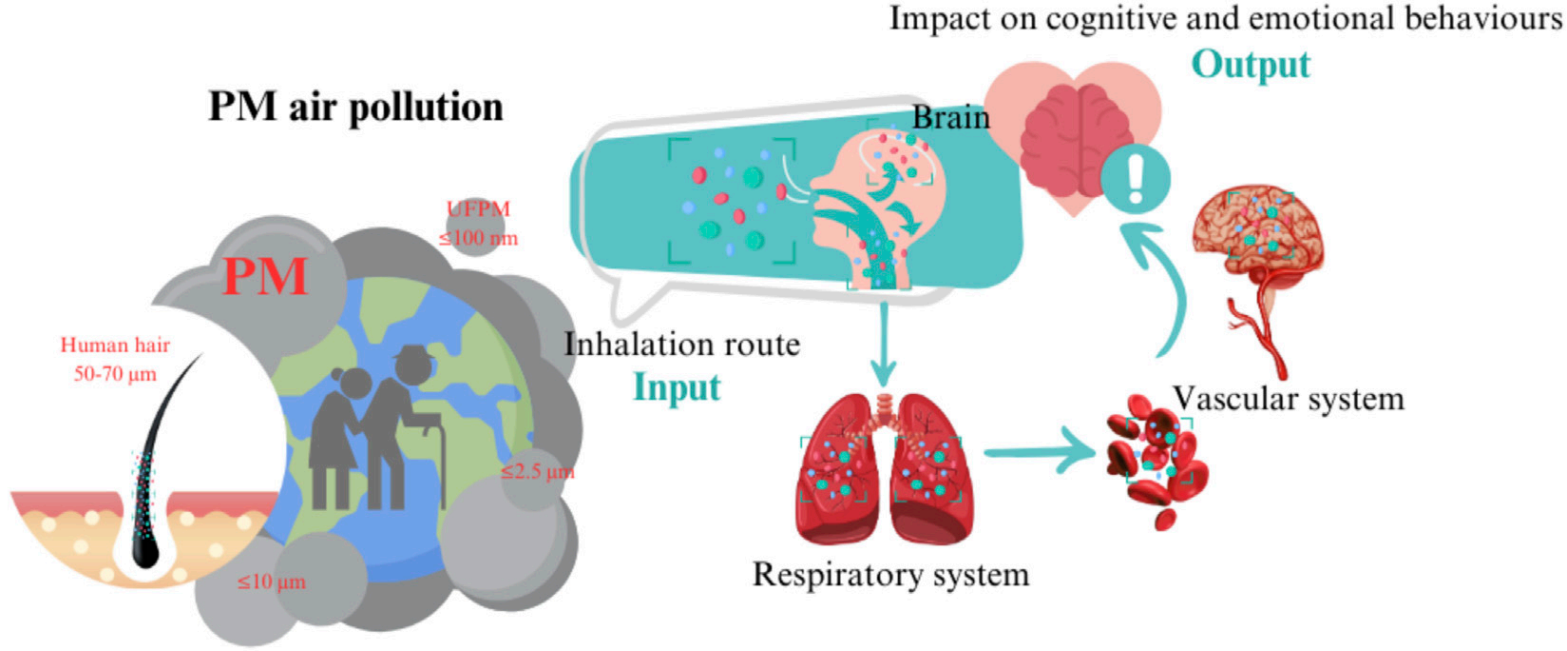
PM originates from a combination of natural and anthropogenic processes. These particles are capable of reaching the CNS and impacting cognitive and emotional functions, especially during aging. The route of access to PM is via inhalation through the nose. PM can (Chen and Kan, 2008) travel directly to the olfactory cortex and other brain regions or (World Health Organization, 2021) PM can be deposited in the lungs. Once it reaches the lungs, PM may also travel through the blood into the brain, causing brain disruption. Figure created with Canva.com.

Road transport, agriculture, power plants, industry and homes are the largest emitters of air pollutants around the world (WHO). Particulate matter (PM) is a widespread and harmful air pollutants (Croze and Zimmer, 2018). PM originates from natural sources (dust, pollen, sea salt, and volcanic eruptions) and human activities (construction, fossil fuel combustion, and industrial processes) (López-Granero et al., 2024). It is also formed by chemical reactions in the atmosphere involving sulfur dioxide, nitrogen oxides, and ammonia, which are affected by temperature and humidity. Since the PM enters the body through the nose until it reaches the brain, the olfactory cortex is one of the main brain areas affected (Ranft et al., 2009; Ajmani et al., 2016). Some authors have demonstrated lower olfactory function in persons who live close to busy highways with heavy traffic (Ranft et al., 2009) and a positive relationship between increased exposure to PM and worse olfactory function (Ajmani et al., 2016) (Figure 1). Therefore, efforts are currently being made to understand the effects of anthropogenic PM derived from diesel vehicle emissions (Fonken et al., 2011). Furthermore, the effects they have on health are serious, due to ease with which they enter the respiratory tract and travel into the brain, making it especially vulnerable (López-Granero et al., 2024; Costa et al., 2019; Costa et al., 2021).

PM is classified according to size: a) PM_10_: coarse particles ≤10 μm, mainly affecting the upper respiratory tract; b) PM_2.5_: fine particles ≤2.5 µm, capable of penetrating deep into the lungs and bloodstream; and c) ultrafine particles: <0.1 µm, with a high surface area to volume ratio, increasing their reactivity and health effects (Costa et al., 2021; Anderson et al., 2012). PM_2.5_, which is known to reduce visibility (haze), poses greater health risks than larger particles (EPA, 2025; Department for Environment and Food and Rural Affairs, 2025). Addressing these sources and properties is crucial for air quality management and public health protection. PM_2.5_ can remain in the air and travel hundreds or thousands of kilometers on air currents, exposing distant populations to pollutants. Studies have shown that long-range transport of PM_2.5,_ spreads pollutants across regional and international boundaries (WHO, 2025). For example, PM_2.5_ from biomass burning, industrial activities, and traffic emissions can be carried by air currents, increasing pollution levels in downwind areas (Manousakas et al., 2021; Lucarelli et al., 2015; Zauli-Sajani et al., 2024; Lin et al., 2022). A study in northern South America found that aerosol transport increased local PM_2.5_ concentrations, increasing health risks such as cardiovascular and respiratory diseases (Velásquez-García et al., 2024). In Kyushu, Japan, researchers found that distant PM_2.5_ sources affected local air quality, highlighting the need for interregional cooperation (Kaneyasu et al., 2014). Similarly, a Mediterranean study found that long-range transport significantly affected PM_2.5_ levels, illustrating how atmospheric dynamics distribute pollutants (Perrone et al., 2013).

Many developed countries, including those in Europe, have failed to maintain urban air pollution levels below the WHO guidelines (World Health Organization, 2017). In 2021, the WHO established primary standards for PM_2.5_ at a 15 μg/m^3^ (24-h average) (WHO, 2023). This problem is illustrated by the fact that this value has been exceeded in many developed countries, surpassing WHO air quality guidelines despite stringent regulations. For instance, the U.S. frequently exceeds PM_2.5_ guidelines in areas like California’s Central Valley and Los Angeles due to vehicle emissions, industrial activity, and geographic factors trapping pollutants (World Health Organization, 2021). In the European Union, cities such as Paris, Milan, and Krakow report PM_2.5_ and NO_2_ levels above the WHO guidelines, driven by traffic congestion, industrial emissions, and solid fuel burning (World Health Organization, 2021). Despite its technological advancements, Japan experiences high ozone and PM_2.5_ levels in cities like Tokyo due to transboundary pollution and domestic industry (World Health Organization, 2021). These examples demonstrate that developed nations still face air quality challenges, requiring continued investment in clean energy, transportation, monitoring systems, and compliance with WHO’s 2021 guidelines (WHO guidelines and monitoring initiatives, accessed via https://www.iqair.com/newsroom/2021-WHO-air-quality-guidelines).

Numerous studies have shown that air pollution is associated with higher susceptibility to respiratory, cardiovascular, and cerebrovascular diseases (Clifford et al., 2016; Zhou et al., 2024) (Figure 1). However, its association with cognitive functioning and impairment requires additional study, as does its relationship with social and emotional status. Some studies have established a connection between air quality, specifically fine particles (PM_2.5_), and cognitive decline in older adults (Tallon et al., 2017). Evidence supports the link between poor air quality, cognitive impairment, and reduced successful aging (Cohen and Gerber, 2017). In fact, air pollution has also been associated with neurodegenerative disorders (Heusinkveld et al., 2016).

Given the potential health risks associated with environmental exposure like air pollution, these risks are particularly prevalent in urban areas due to gaseous pollutants and PM (Gatto et al., 2014). Thus, the connection between cognitive decline and exposure to neurotoxic ambient air pollution in urban areas (Gatto et al., 2014) is further reinforced by a series of studies published in recent years, which also highlight the relationship between urban environments and elevated levels of oxidative stress in the elderly (Sánchez-Rodríguez et al., 2006). Air quality appears to influence biological processes that directly affect cognition (Midouhas et al., 2018) and emotion (Lim et al., 2012; Power et al., 2015; Sass et al., 2017) by inducing neuroinflammation and oxidative stress (Block and Calderón-Garcidueñas, 2009; Calderón-Garcidueñas et al., 2004). Some studies have indicated that air pollutants can penetrate the blood-brain barrier, leading to systemic inflammation that negatively affects the central nervous system (Block and Calderón-Garcidueñas, 2009).

As mentioned, exposure to environmental pollution has been associated with a range of emotional alterations, such as depression, anxiety, and psychological stress (Lim et al., 2012; Power et al., 2015; Sass et al., 2017). Depression, the emotional factor that is most commonly associated with cognitive decline, has attracted significant academic attention. In geriatric populations, depression often co-occurs with measurable cognitive decline (Modrego and Ferrández, 2004; Rapp et al., 2011). Geriatric depression is considered a risk factor for Alzheimer’s disease (AD) (Rapp et al., 2011) and has been shown to increase the risk of dementia (Barnes et al., 2012; Ownby et al., 2006). Depression is also associated with cognitive decline and the onset of mild cognitive impairment (Feola et al., 2013; Wilson et al., 2014). However, these distinct pathologies are frequently confused (Dillon et al., 2014). Furthermore, a high prevalence of attention deficits has been linked to poor air quality (Siddique et al., 2011; Oudin et al., 2016; young and bok, 2017). Additionally, impaired autonomic and cognitive functions are predictors of impulsive-compulsive behavior at the time of a Parkinson’s disease (PD) diagnosis (Ricciardi et al., 2018).

According to several definitions, the process of human aging is complex and occurs in the biological, psychological and social spheres (Dziechciaż and Filip, 2014). A one-sided approach to aging would be incorrect. We raise the following question: Can air quality modulate cognitive, emotional, and social behaviors during aging? The aim of the present review was to deepen our understanding of behavioral consequence-dependent air quality during the aging process and the role of social health. In addition, it initiated a debate at the social and governmental levels regarding policies established by countries to address this public health issue.

## 2 The relevance of pollution-free residence for successful aging

The urban population is increasing rapidly worldwide. As cities expand, environmental quality, particularly urban air pollution, has become an increasingly critical factor in public health (Salim et al., 2013). Few studies have focused on identifying the predictive factors of cognitive decline in healthy elderly adults (Baumgart et al., 2015; Nguyen et al., 2002). Among these factors, there are variables centered on areas such as lifestyle and intellectual and social activities (Verghese et al., 2006). Following this argument, the Lancet Commission published a report adding new evidence to include pollution as a modifiable risk factor for dementia, including traumatic brain injury, high blood pressure, obesity, hearing loss, depression, diabetes, lower education, physical inactivity, smoking, alcohol abuse, social isolation, and pollution (Livingston et al., 2020).

It seems that the location of residence, whether rural or urban, and its inherent relation to environmental toxicity, pollution, and social conditions in these areas, could be considered as a predictive factor for healthy aging. Therefore, environmental pollution and the lack of socialization linked to urban environments deserve more attention in the study of risk factors of cognitive decline and emotional alterations (Gatto et al., 2014; Parra et al., 2022). In fact, some authors have already identified social relationships as a protective factor against aging (Tobiasz-Adamczyk and Zawisza, 2017). Tobiasz-Adanzczyk and Zawisza found an association between social activities and interactions and a better positive self-evaluation of health among elderly people living in rural environments in Poland (Tobiasz-Adamczyk and Zawisza, 2017). Furthermore, being married has been shown to be a protective factor against the effects of pollution, especially in the male population in South Korea (Kim et al., 2020).

Some studies have shown significant cognitive decline in relation to long-term exposure to urban pollution indices in different countries (Tan et al., 2023; Trushna et al., 2021; Weuve et al., 2012; Zeng et al., 2010; Zhang et al., 2024a; Tuffier et al., 2024). Air pollution is also associated with neurodegenerative conditions (Dimakakou et al., 2018; Shin et al., 2018; Yuchi et al., 2020). For example, it has been associated with a 2-year excess decline in cognitive function in relation to each 10 mg/m^3^ increase in long-term PM exposure in older women living in 11 US states, (Weuve et al., 2012). Other authors found similar results in women recruited from 24 states and Washington, DC, United States (Younan et al., 2022). In Brazil, Garbaccio et al. (2018) evaluated the quality of life of elderly people and concluded satisfactorily with good health in the cognitive processes of participants living in a rural Brazilian context. The favorable results derived from these studies merit discussion on a better redistribution of the population in favor of rural areas.

**FIGURE 2:**
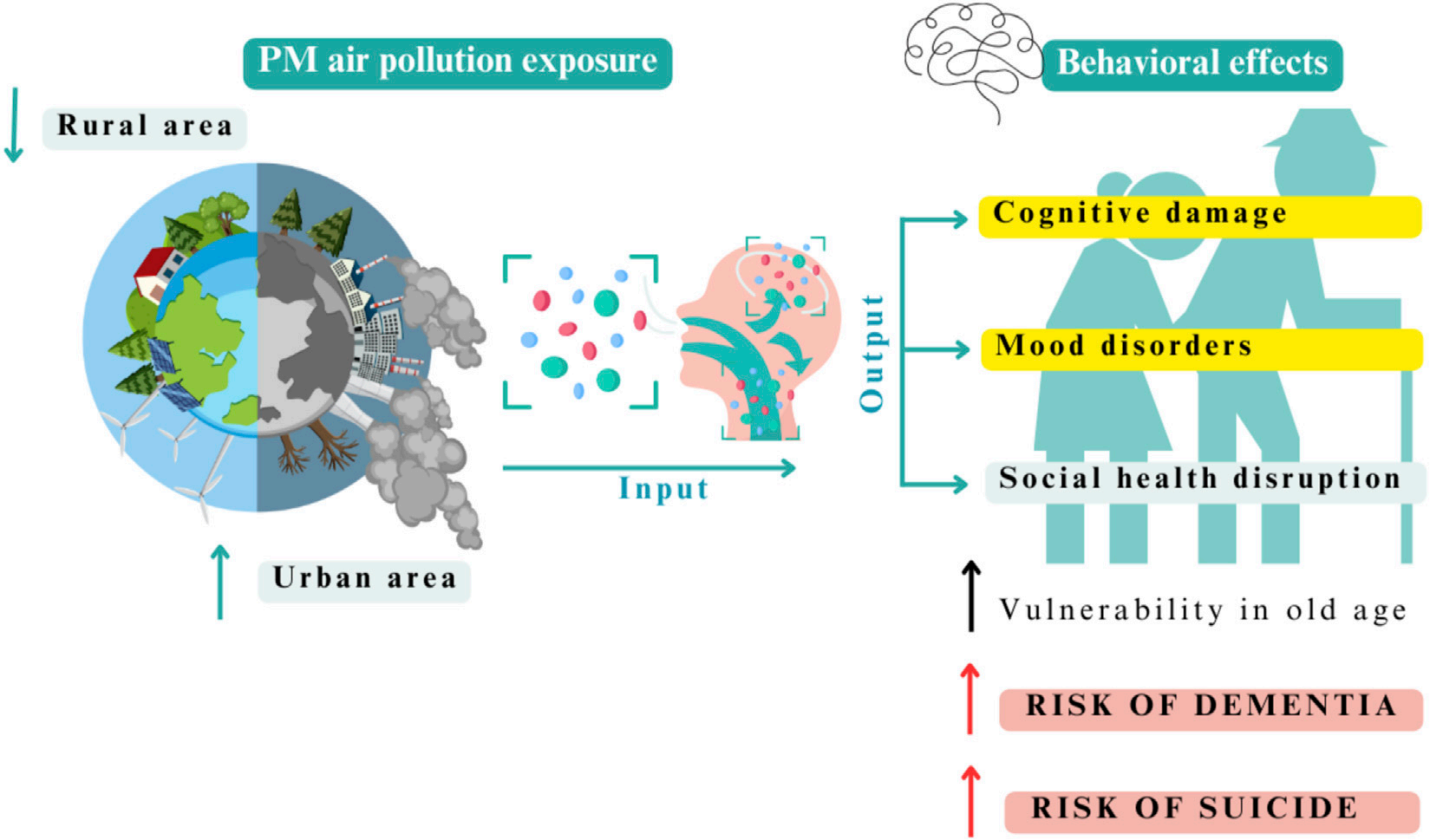
Overview of PM air pollution exposure and behavioral effects on human aging. In urban areas, the exposure to inhaled PM is high, leading to cognitive damage, mood disorders, and social health disruptions. The consequence is a higher vulnerability in older people with an increased risk of dementia and suicidal behavior. Figure created with Canva.com.

A consensus exists about the damage caused by pollution in urban areas in several cities worldwide (Gatto et al., 2014; Parra et al., 2022). However, other factors indirectly associated with air pollution exposure may contribute to the observed differences in cognitive abilities, such as socioeconomic status and emotional status. Exposure to environmental pollution is related to a range of symptoms such as depression, anxiety and psychological stress (Power et al., 2015; Lim et al., 2012; Sass et al., 2017; Tan et al., 2023; Trushna et al., 2021). Depression is also associated with cognitive decline and the incidence of mild cognitive impairment (Tan et al., 2023; Trushna et al., 2021; Tan et al., 2023; Trushna et al., 2021; Tan et al., 2023; Trushna et al., 2021; Tan et al., 2023; Trushna et al., 2021) and even geriatric depression has been considered a risk factor for AD (Rapp et al., 2011). These distinct pathologies are frequently confused in older adults (Dillon et al., 2014) (Figure 2).

## 3 Air pollution and cognitive functioning in elderly people

With the rapid aging of the global population, it is estimated that this sector of the population increases annually between 12% and 22%, and within this group approximately 14% of adults aged 60 and over live with mental disorders (WHO, 2023). Air pollution is one of the factors associated with harmful effects on the central nervous system (CNS) is air pollution.

PM can reach other brain areas and cause cognitive dysfunctions (Table 1). Long-term exposure to PM_2.5_ has been shown to be strongly linked to impaired cognitive function in older Americans (exposure to PM_2.5_ also produces long-term mood disorders that have related pathways to affect as well cognition) (Tallon et al., 2017; Petkus et al., 2021). Inhalation of PM_2.5_ particles is associated with poor performance according to the Mini-Mental State Examination (MMSE) among the elderly in the US (Gatto et al., 2014; Parra et al., 2022), rural and suburban China (Ji et al., 2020; He et al., 2020; Wang et al., 2020) and Korea (Lee et al., 2022). Each 10 μg/m^3^ increase in exposure is estimated to be equivalent to two additional years of cognitive decline due to aging (Power et al., 2011). According to previous studies, a relationship between PM exposure and poor cognitive function (Cho et al., 2023; Ogurtsova et al., 2023; Kulick et al., 2020; Gui et al., 2024). A longitudinal study on aging in Taiwan (TLSA) established a relationship between exposure to PM with an aerodynamic diameter <10 μm (PM_10_) and cognitive impairment in older adult populations (Lo et al., 2019). This study demonstrated that co-exposure to ambient PM_10_ and O_3_ may deteriorate cognitive function to a greater extent in older adults. It is a fact that PM is able to damage the cognitive function by itself or along to others air pollutants such as O_3_ (Gatto et al., 2014; Gao et al., 2022).

**TABLE 1:** Impact of PM exposure on older adults: key cognitive impairments and increased risk of dementia [Alzheimer’s Disease (AD), Parkinson’s Disease (PD), and Vascular Dementia (VD)] associated with PM exposure. Refer to Figure 6 for details on the geographical distribution.

References	Population and molecule origin	PM molecule size and measurements	Main affectations observed
Ailshire et al. (2014)	United States: 13,996 men and women over 50 years (mean = 64)	PM_2.5_: concentrations were derived using 24-h daily means reported by monitors within a 60-km radius of each census tract centroid	Episodic memory
Ailshire and Clarke (2015)	United States: 780 men and women over 55 years	PM_2.5_: national tract-level pollution measures were derived from data collected in the Environmental Protection Agency’s Air Quality System (AQS) and were calculated based on air monitoring data sites within a 60-km radius of the respondent’s tract centroid	General cognitive function
Ajmani et al. (2016)	United States: 2,221 men and women over 57 years (mean = 67.9)	PM_2.5_: geographic Information Systems (GIS)-based spatio-temporal models predicting monthly PM_2.5_ concentrations	Olfactory function
Cho et al. (2023)	South-Korea: 640 men and women over 50 years (mean = 67.4)	PM_10_ y PM_2.5_: were estimated by using the universal kriging model and regulatory air quality monitoring data	General cognitive function
Gatto et al. (2014)	United States: 1,496 men and women (mean = 60.5)	PM_2.5_: employing a GIS-based system, yearly air pollution exposure assignments were derived from measured ambient air quality data spatially mapped	Verbal learning
Gui et al. (2024)	China: 176,345 men and women over 60 years (mean = 72.38)	PM_2.5_ y PM_10_: daily mean concentrations of PM were directly downloaded from the China High Air Pollutants (CHAP, available at: https://weijing-rs.github.io/product.html) dataset.	General cognitive function
Jung et al. (2015)	Taiwan: 95,690 men and women over 65	PM_2.5_ y PM_10_: hourly, data were available from 70 Taiwan Environmental Protection Agency (EPA) monitoring station on Taiwan’s main island from 2000 through 2010	Dementia Risk (AD)
Lee et al. (2022)	South-Korea: 4,175 men and women over 50 years (mean = 67.8)	PM_2.5_ y PM_10_: air quality parameters and meteorological databases were established using the Community Multiscale Air Quality (CMAQ) model	General cognitive function
Lo et al. (2019)	Taiwan: 2,241 over 65 years	PM_10_: levels from 75 monitoring stations constructed by the Taiwan Environmental Protection Administration (TEPA)	General cognitive function
Ogurtsova et al. (2023)	Germany: 2,554 men and women over 45 years (mean = 63.2)	PM_2.5_, PM_10_, PM_2.5 abs_ and PN_acc_ (accumulation mode particle number): the study area included about 600 km^2^. PM were estimated using the European Study of Cohorts for Air Pollution Effects (ESCAPE) land use regression (LUR) model	Verbal learning and memory
Parra et al. (2022)	UK: 187,194 men and women over 60 years (mean = 64.1)	PM_2.5_, PM_10_, PM_2.5–10_, PM_2.5absorbance_: measures were provided by the Small Area Health Statistics Unit (http://www.sahsu.org/) as part of the BioSHaRE-EU Environmental Determinants of Health Project (http://www.bioshare.eu/) of which the UK Biobank is a collaborator	Dementia Risk (AD and VD)
Ran et al. (2021)	China: 57,775 men and women over 65 years	PM_2.5_: the annual PM_2.5_ concentration during 1998–2011 at each participant’s residential address was assessed by a satellite-based model	Dementia Risk (AD and VD)
Ranft et al. (2009)	Germany: 399 women over 68 years (mean = 74.1)	PM_10_: provided by monitoring stations distributed over the Ruhr district in an 8-km grid and a geo-information system was used to determine ambient air pollution exposure on a small scale	Olfactory function
Salinas-Rodríguez et al. (2018)	Mexico: 7,986 over 60 years	PM_2.5_: estimation of PM_2.5_ concentrations was done at aggregated level using the Census Tract Units (named Basic Geostatistical Areas in Mexico, AGEB by its Spanish acronym), and assigning this exposure to the households residing in those areas	Verbal learning and memory
Schikowski et al. (2015)	Germany: 789 women over 55 years (mean = 73.4)	PM_2.5_, PM_2.5abs_ and PM_10_: were estimated by land-use regression (LUR) models according to the ESCAPE study (European Study of Cohorts for Air Pollution Effects)	General cognitive function (Visuospatial domain) Dementia Risk (AD, ApoE ε4)
Shin et al. (2018)	United States: 2,194,519 men and women over 55 years (mean = 67)	PM_2.5_: concentrations were derived from high-resolution satellite observations for the years 1998–2012, based on the Moderate Resolution Imaging Spectroradiometer of the National Aeronautics and Space Administration’s Terra/Aqua satellite	Dementia Risk (PD)
Shim et al. (2023)	South-Korea: 1,436,361 men and women over 65 years (mean = 70.9)	PM_10_: air Korea (www.airkorea.or.kr) was used to collect data from the region-specific sites. The data were sent to the National Ambient Air Monitoring Information System (NAMIS)	Dementia Risk (VD)
Tallon et al. (2017)	United States: 3,377 men and women over 57 years (mean = 72.38)	PM_2.5_: concentrations were estimated on a 6-km (km) grid covering the conterminous United States from a set of five spatio-temporal generalized additive mixed models	General cognitive function
Wang et al. (2020)	China: 13,324 over 65 (mean = 82.4)	PM_2.5_: were derived from a remote-sensing grid data by the Institute of Atmospheric Physics, University of Dalhousie	General cognitive function
Wu et al. (2015)	Taiwan: 249 AD, 125 VD and 497 healthy controls Both over 60 years (mean = 79.1, 79.9 and 72.9 respectively)	PM_10_: ambient monitoring data were obtained from Taipei–Keelung metropolitan area including 24 monitoring stations from the Department of Taiwan Air Quality Monitoring Network, Environmental Protection Administration (EPA)	Dementia Risk (AD and VD)
Younan et al. (2020)	United States: 998 women aged 73 years	PM_2.5_: was estimated using the Bayesian maximum entropy (BME)-based spatiotemporal modelling approach, integrating nationwide monitoring data from both the US Environmental Protection Agency (EPA) Air Quality System (AQS) and the output of chemical transport models, entitled Community Multiscale Air Quality	Episodic memory (Immediate recall and new learning) Dementia Risk (AD)
Yuchi et al. (2020)	United States: 678,000 men and women over 45 years	PM_2.5_: land-use regression (LUR) models specific to Metro Vancouver were applied to estimate exposures to PM	Dementia Risk (PD)
Zhang et al. (2024)	UK: 155,828 men and women over 60 years (mean = 64.09)	PM_2.5:_ concentrations were determined using the Land Use Regression (LUR) models developed by the European Study of Cohort and Air Pollution Effects (ESCAPE) and correlated with the geocodes baseline residential address of the participants in the UK Biobank	Dementia Risk (AD and VD)

Some studies have indicated that a specific cognitive subdomain is affected by PM exposure, not only by general cognitive status (Table 1). Specifically, in Germany, a study with a sample comprised of women older than 75 years indicated that those who lived near a busy road showed poorer performance in executive functions compared to those women who resided further from busy highways with heavy traffic (Ranft et al., 2009). Gatto and collaborators (2014) using a sample of 1,500 people with an average age of 60 years, found a reduction in verbal learning in US (Gatto et al., 2014; Parra et al., 2022). PM_2.5_ has also been found to strongly associate with a higher error rate on orientation and working memory tests in adults 55 years of age and older (Ailshire and Clarke, 2015) and reduced episodic memory compared to those with less exposure to PM_2.5_ (Younan et al., 2022; Ailshire and Crimmins, 2014). In addition, exposure to PM has been associated with lower scores on immediate verbal memory (Ogurtsova et al., 2023) and semantic verbal fluency (Salinas-Rodríguez et al., 2018).

Therefore, these studies have associated the exposure to PM with deficits in memory, working memory, episodic memory, verbal learning, memory, and attentional processes (Table 1) (López-Granero et al., 2024; Gatto et al., 2014; Ogurtsova et al., 2023; Younan et al., 2020; Yao et al., 2022a; Tonne et al., 2014; Wurth et al., 2018). It is noteworthy that working memory and attention are essential for normal cognitive development as well as being one of the first cognitive processes affected during aging. Working memory is a cognitive system that temporarily holds information for processing. Its function is crucial for any cognitive competence, such as learning, reasoning, problem solving and understanding language (Rivas et al., 2019; Vuontela et al., 2003). On the other hand, attention is a basic function required for higher cognitive abilities (e.g., executive function or memory) and involves different processes, such as selective attention to a particular source of stimulation or voluntary control (Anderson, 2002). Attention is also essential for controlling executive function. Inhibitory control deficits are characterized by the presence of impulsive behaviors, being a trait which appears to be present in various neuropsychopathological and neurodegenerative disorders (Robbins et al., 2012). It has also been indicated that poor cognitive performance and anxiety symptoms can predict the development of impulsive-compulsive behavior at the time of PD diagnosis (Ricciardi et al., 2018). In this sense, we highlight that the high prevalence of attention deficit has been associated with poor air quality in younger people (Tan et al., 2023; Trushna et al., 2021; Oudin et al., 2016; Young and Bok, 2017); however, it has not been studied in aging people, even when the interest in impulsivity is based on the theory that self-control could be related to measures related to longevity is currently on the rise (Strulik, 2019; Cohen and Gerber, 2017).

## 4 Air pollution and dementia

**FIGURE 3:**
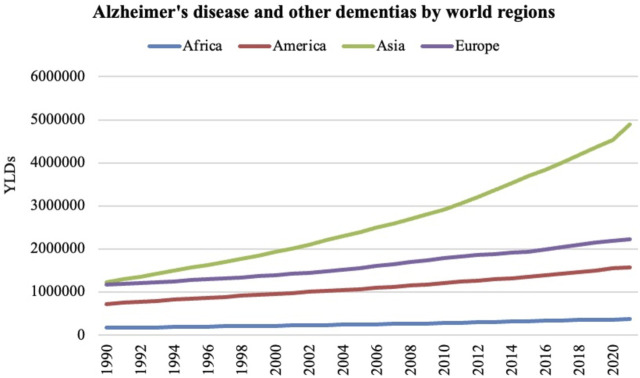
The number of years lived with disability (YLDs) in the population aged over 70 years in relation to AD and other dementias from 1990 to 2021 in Africa, America, Asia, and Europe. Institute for Health Metrics and Evaluation (IHME). GBD Compare Data Visualization. Global Burden of Disease (GBD) Study 2021. Seattle, WA: IHME, University of Washington, 2023. Available from http://vizhub.healthdata.org/gbd-compare. (Accessed 12/19/2024).

According to the WHO, the number of cases of dementia worldwide is increasing every 20 years by double the number of cases, taking into account the growth of the elderly population around the world (United Nations: World Urbanization Prospects, 2010), particularly in developing countries. In this regard, Figure 3 shows a comparative graph for increasing cases of AD and related dementias in Africa, America, Asia and Europe from 1990 to 2021. This graph shows how the number of years lived with disability (YLDs) in the population aged > 70 years in relation to AD and other dementias has increased over time. This trend is most prominent in regions of America, Asia, and Europe, with comparatively fewer cases reported in Africa.

The 2017 Lancet Commission on Dementia Prevention, Intervention, and Care produced a list of possible risk factors for dementia including air pollution (Livingston et al., 2017). The 2018 Lancet Commission on Pollution states that a concurrent relationship is being built, specifically between fine PM and dementia in older people, thus requiring further research to examine emerging causal links (Landrigan et al., 2018).

In recent years, several human studies have demonstrated a link between air pollution exposure and dementia pathologies such as AD and PD (Table 1) (Costa et al., 2020; Mohammadzadeh et al., 2024; Peters et al., 2019). These findings raise the hypothesis that inhalation of particles (Calderón-Garcidueñas et al., 2004) negatively affects the brain, involving the activation of an inflammatory response and increasing production and deposition of Aβ peptides (Block and Calderón-Garcidueñas, 2009). A high incidence and risk of AD and PD dementias has been established for people exposed to PM ambient pollution and other air contaminants (such as NO_2_, CO, and O_3_ to name a few), around the world (e.g., Taiwan, Germany, Sweden, the United States, China and the UK) in the elderly (Parra et al., 2022; Braithwaite et al., 2019; Chang et al., 2014; Jung et al., 2015; Kasdagli et al., 2019; Schikowski et al., 2015; Wu et al., 2015; Ran et al., 2021; Wu et al., 2022). In a health and environment cohort study on a sample in the Ontario population (Chen et al., 2016) it was observed that exposure to PM_2.5_ was associated with a high incidence of dementia. In two other cohort studies involving 96,000 and 30,000 adults in Taiwan, exposure to PM_2.5,_ was associated with a 54%–212% increase in the incidence of dementia (Chang et al., 2014; Jung et al., 2015). In another study carried out in the UK, positive associations were found between exposure to PM_2.5,_ and the risk of all-cause dementia (Zhang et al., 2024b).

The social security coverage program administered by the United States government, which provides medical care to all people over 65 years of age known as Medicare, in one of the reviews of its users found data that for each increase of 1 μg/m^3^ at the annual concentration of PM_2.5_ citywide, there was an adjusted risk ratio (HR) of 1.08 for dementia (Kioumourtzoglou et al., 2016). Similar results have been found in other countries such as the UK, where samples from the UK Biobank were used to follow 155,828 elderly people aged 60 years and without a diagnosis of dementia for 12 years. Increases of 1 μg/m^3^ in ambient PM_2.5_ were found to have an adjusted hazard ratio (HR) of 1.07 for all-cause dementia risk (Zhang et al., 2024b). In general, it increases the risk of AD (Parra et al., 2022; Shim et al., 2023), PD (Shin et al., 2018; Yuchi et al., 2020) and vascular dementia (VD) (Parra et al., 2022; Shim et al., 2023) (Table 1).

Some authors have demonstrated an association between air pollution and respiratory and cardiovascular morbidity and mortality (Chen and Kan, 2008; Franchini and Mannucci, 2009; Ghio et al., 2012; Götschi et al., 2008; Pelucchi et al., 2009). VD is the second most common pathophysiological process (20%) after AD (Enhancing care, Advancing Health Science, 2025). Cardiovascular disease (CVD) is also known to affect cognitive function in later years (O’Brien, 2006; Rundek et al., 2022; Kalaria et al., 2016), and vascular and metabolic risk factors, including high blood pressure, obesity, diabetes, and stroke (Rosamond et al., 2008) are all inversely associated with cognitive function in middle-aged and older adults (Leritz et al., 2011).

It is worth mentioning that dementia and cognitive impairment, unlike cardiovascular disease, have no current treatment options (Livingston et al., 2020; Livingston et al., 2017) and should be considered an even higher risk to the population if the link between PM and cognitive decline is shown to be strong.

## 5 Air pollution and depression symptoms in elderly people

**FIGURE 4:**
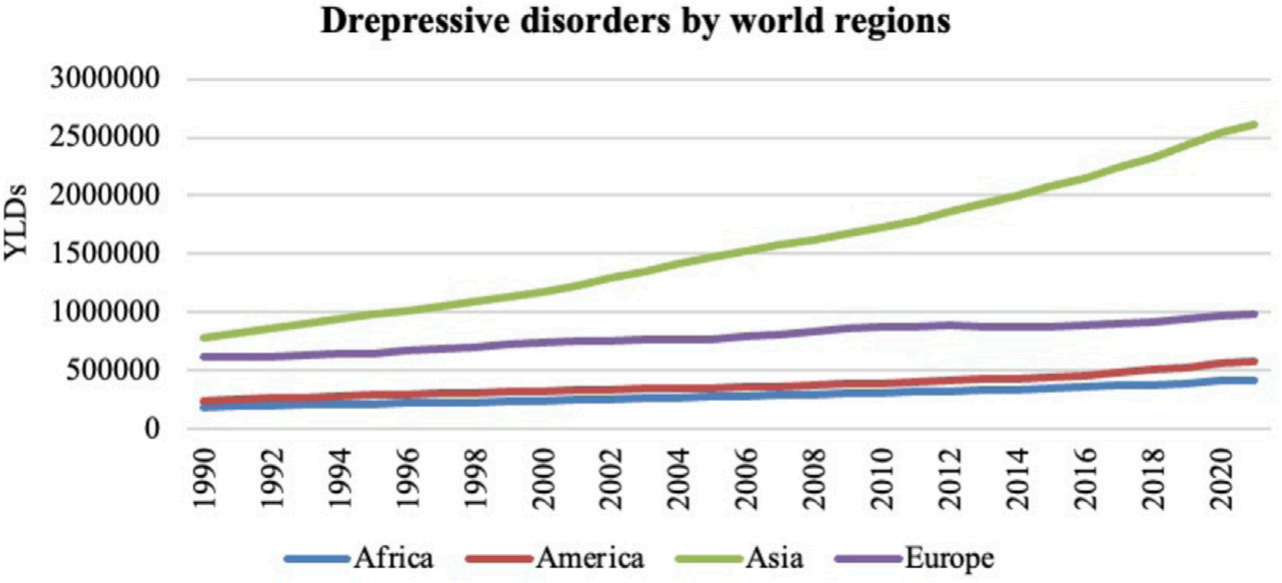
The number of years lived with disability (YLDs) in the population aged over 70 years in relation to depressive disorders from 1990 to 2021 in Africa, America, Asia and Europe. Institute for Health Metrics and Evaluation (IHME). GBD Compare Data Visualization. Global Burden of Disease (GBD) Study 2021. Seattle, WA: IHME, University of Washington, 2023. Available from http://vizhub.healthdata.org/gbd-compare. (Accessed 12/19/2024).

A wealth of research shows that air pollution influences emotional responses such as happiness, anxiety, depression symptoms, life satisfaction, stress, sadness, pain, powerlessness, and nervous and mental disorders. These emotional responses to air pollution have been documented worldwide, including Australia, Europe, the United States, Latin America, Canada, and China (Altuğ et al., 2020; Barnett et al., 2018; Lu, 2020; Mendoza et al., 2019; Wang et al., 2019). One of the main emotional consequences of air pollution is depression or depressive symptoms, which have been increasingly observed in recent years. Figure 4 illustrates this rise in cases in Africa, America, Asia, and Europe from 1990 to 2021 among older adults. According to the World Health Organization (World Health Organization, 2017), depression is one of the most common mental health issues affecting more than 300 million people worldwide. A meta-analysis published in 2018 showed a statistically significant association between air pollution and depressive symptoms in older adults (Barnett et al., 2018) (Table 2).

**TABLE 2:** Impact of PM exposure on older adults: key mood disorders associated with PM exposure. Refer to Figure 6 for details on the geographical distribution.

References	Population and molecule origin	Molecule size and measurements	Mood disorders observed
Altuğ et al. (2020)	Germany: 821 older women over 65 years (mean = 73.5)	PM_10_, PM_2.5_, PM_coarse_, PM_2.5abs_: were assigned to the participating women’s home addresses by land-use regression (LUR) models according to the ESCAPE study (European Study of Cohorts for Air Pollution Effects)	Depressive behavior (diagnosis or symptoms)
Gu et al. (2020)	China: 14,772 men and women over 15 years (mean = 32.67)	PM_2.5:_ was measured by the annual average concentration	Depressive behavior Anxious behavior
Kim et al. (2010)	Republic of Korea: 4,341 cases of suicide. Men and women over 35 years	PM_2.5_ y PM_10_: hourly mean concentrations were measured at 13 sites in Seoul. Data were provided by the Ministry of Environment and the Seoul Metropolitan Government. Meteorological data	Suicide Risk
Kim et al. (2020)	South Korea: 2,729 men and women over 50 years (mean = 69.58 and 68.22 for men and women respectively)	PM_2.5_ y PM_10_: were obtained from the prediction model developed by Kim and Song (2017)	Depressive behavior
Kovess-Masféty et al. (2000)	Belgium, France, Germany, Italy, the Netherlands and Spain: 21,425 men and women over 18 years (mean = 48.59 and 47.79 in rural and urban areas respectively)	The rural population is defined as those living in towns with fewer than 10 000 inhabitants, and the urban population is defined as those living in towns or cities with 10 000 or more inhabitants	Urbanicity depressive behavior anxious behavior
Lim et al. (2012)	Korea: 537 men and women over 60 years (mean = 71)	PM_10_: data from the Research Institute of Public Health and Environment [Ministry of Environment (MOE) 2009, 2010, 2011]. For Seongbuk-Gu residents (90% of the study population), one monitor, which was centrally located in Seongbuk-Gu, was used as a proxy for individual exposure to ambient pollutants. For other residents, the nearest monitoring site to their residential address was used as a measure of air pollution concentrations	Depressive behavior
Min et al. (2018)	South-Korea: 26,5749 men and women over 20 years	PM10: data obtained from the National Ambient Air Monitoring Information System (NAMIS) (Korea Environment Corporation, 2017). The NAMIS provided daily air pollution data from community-based monitoring sites (267 sites in 2002) that were available in real-time through the organizations’ website (Korea Environment Corporation, 2017)	Suicide Risk
Motoc et al. (2023)	Netherlands: 1,785 men and women over 57 years (mean age 70 and 70.2 for depression and anxiety sample respectively)	PM_2.5_: were calculated by the Institute for Risk Assessment Sciences as part of the European Study of Cohorts for Air Pollution Effects (ESCAPE-project) using land-use regression models for the year 2009	Urbanicity anxious behavior
Petkus et al. (2021)	United States: 1,583 women over 80 years	PM_2.5_: were estimated using regionalized universal kriging models, which were based on US Environmental Protection Agency (EPA) monitoring data	Depressive behavior
Pun et al. (2017)	United States: 4,008 men and women over 57 years (mean = 69 and 71 in the wave 1 and 2 respectively)	PM_2.5_: daily PM_2.5_ estimates on a 6-km grid covering the conterminous United States were obtained from a set of five spatio-temporal generalized additive mixed models (GAMMs)	Depressive behavior Anxious behavior
Tan et al. (2023)	China: 5,717 men and women over 65 years (mean = 71.85)	PM_2.5_ y PM_10_: based on the SHSS geo-coded address of the administrative village in the 5 years prior to the survey within a radius of 3 km for. Concentrations were collected from the open-access ChinaHighAirPollutants (CHAP) datasets	Depressive behavior Anxious behavior
Wang et al. (2019)	China: 20,861 men and women over 18 years (mean = 44.83)	PM_2.5_: data were derived from the Airborne Fine Particulate Matter and Air Quality Index website (AFPMAQI, 2016). PM_2.5_ data for 2016 were extracted from a nationwide measurement network comprising 1,613 monitoring stations distributed across China	Depressive behavior

This association has been replicated in epidemiological and experimental studies with similar results. In 2019, Zhang and Wang (Zhang and Wang, 2019) found that high annual ground-level PM_2.5_ concentrations had significant negative effects on the average level of happiness in China with cross-sectional data. In addition to the Chinese population, some authors found that a higher concentration of PM_2.5,_ significantly increased the prominence of the four negative emotions such as nervousness, depression, powerlessness, and restlessness (Gu et al., 2020). Additionally, Buoli et al. (2018) conducted a review with interesting results: long exposure to PM_2.5_ pollutant increased depressive symptoms (d effect size: 0.05–0.81 for each increase of 5–10 μg/m^3^ of air pollutant concentrations) (Buoli et al., 2018). Another review and meta-analysis study (Braithwaite et al., 2019) indicated that higher long-term exposure (>6 months) to PM_2.5,_ was associated with higher odds of depression. Recently, some authors found that air pollution parameters, such as PM_10_ and PM_2.5,_ were significantly and positively associated with depressive symptoms, even after adjustment for a German sample of elderly women (Altuğ et al., 2020). In addition, this study indicated that living within 100 m of a major road was positively associated with depression. Lim et al. (2012) conducted a study on elderly Chinese people and found that PM concentrations were significantly and positively related to depression. A systematic review and meta-analysis conducted in 2018 (Barnett et al., 2018) showed that air pollution, among other neighborhood characteristics, was positively related to depressive symptoms in the elderly population. This study evaluated a total of 73 articles with data from Asia, Europe, United States, and Oceania. Recently, two systematic reviews were published (Braithwaite et al., 2019; Cao et al., 2024). Both, which include a total of 22 and 25 papers, respectively, find associations between long-term exposure to PM_2.5_ and depression.

Overall, some of the aforementioned studies emphasized that the relationship between air pollution and mood disorders is remarkably different in rural and urban areas (Table 2). Similarly, Kovess-Masféty et al. also indicated that urbanicity seemed to be linked to a higher risk of mental health disorders, particularly depressive disorders (Kovess-Masféty et al., 2000). This finding is part of The European Study of the Epidemiology of Mental Disorders (ESEMeD 2000 study), which collected data from 6 European countries: Belgium, France, Germany, Italy, the Netherlands, and Spain. In Belgium, women in rural areas had fewer emotional disorders. Therefore, it seems that subjects living in metropolitan areas are more vulnerable to long-term PM_10_ exposure than those living in non-metropolitan areas (Young et al., 2018). It is worth mentioning that air pollution seems to trigger suicidal behavior; some authors have even indicated that air pollution exposure is associated with an increased risk of suicide in adults (Braithwaite et al., 2019; Young et al., 2018) (see Table 2). Kim and collaborators suggest that increased ambient PM associated to a higher suicide risk is more pronounced in persons 36–64 years of age (Enhancing care, Advancing Health Science, 2025). There are still insufficient studies that addressed the relationship between air pollution and suicide risk in the elderly.

In addition, it seems that air pollution contributes to depressive symptoms in older adults through feelings of loneliness (Zhang and Wang, 2019). Similarly, urbanicity was significantly associated with increased depressive symptoms, mediated by loneliness, physical activity, social support and air pollution (Pun et al., 2019). Social interactions seem to play a crucial role in shaping the overall quality of life of adults, particularly older adults (Scharf and Jong Gierveld, 2008; Bowling, 2005). Evidence indicates that limited or poor social connections can elevate the risk of dementia (Gardener et al., 2021). In addition, it has been shown that the probability of mortality increases by 30% in situations of loneliness, social isolation and living alone, so that, loneliness and isolation are considered predictors of physical, mental and psychosocial health problems in both rural and urban areas (Ibarra et al., 2020). Several studies underscore the advantages of aging in rural communities, such as robust social networks, enhanced social integration, and healthy and safe environments (Winterton and Warburton, 2012).

As well, depression is often associated with a decrease in overall wellbeing, affecting emotional, psychological, and social dimensions (Steptoe et al., 2015). Accordingly, air pollution may impair population health by influencing people’s subjective wellbeing (Li et al., 2019). Alternatively, as proposed by others, air pollution reduces subjective wellbeing by affecting physical health or reducing social contact with people outdoors, which indirectly affects subjective wellbeing (Zhang and Wang, 2019). In the study by Benavente Cuesta and Quevedo Aguadode (2019) on the Autoperception of Health, Quality of Life and Psychological Wellbeing among older people, a significant relationship was found between self-perception of health and the area in which people reside (rural and urban) (Benavente Cuesta and Quevedo Aguadode, 2019). The perception that the two groups have about their interpersonal relationships is attributed in this study to the phenomenon of depopulation experienced in rural areas in recent decades (Benavente Cuesta and Quevedo Aguadode, 2019).

## 6 Air pollution and anxiety emotion in elderly people

**FIGURE 5:**
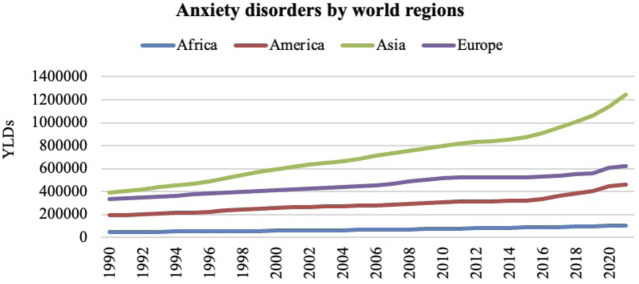
The number of years lived with disability (YLDs) in the population aged over 70 years in relation to anxiety disorders from 1990 to 2021 in Africa, America, Asia and Europe. Institute for Health Metrics and Evaluation (IHME). GBD Compare Data Visualization. Global Burden of Disease (GBD) Study 2021. Seattle, WA: IHME, University of Washington, 2023. Available from http://vizhub.healthdata.org/gbd-compare. (Accessed 12/19/2024).

The effects of air pollution exposure on anxiety in older adults have not been well studied, despite the increasing diagnosis of anxiety disorders in the last 30 years around the world (see Figure 5). Negative emotions, such as nervousness, powerlessness, anxiety, and annoyance, have been linked to air pollution (Table 2). A few studies in the US have shown that an increase in PM_2.5_ is linked with anxiety symptoms, with the largest increase in the 180-day moving average during aging (Pun et al., 2017). In China, Tan and collaborators, in 2023 also found positive associations between exposure to PM_2.5,_ PM_10_ and an increased prevalence of pain/discomfort and anxiety/depression mediated by higher socioeconomic status (affluent, urban, and higher level of education) (Tan et al., 2023).

A recent review (Lu, 2020) showed that exposure to PM_2.5_ in elderly women predicted increased anxiety symptoms. In addition, a systematic review and meta-analysis by Braithwaite team in 2019 reported significant positive associations between long-term exposure to PM_2.5_, and anxiety (Braithwaite et al., 2019). Higher concentrations of PM_2.5,_ have been found to increase the negative emotions of nervousness and powerlessness in the Chinese population (Gu et al., 2020). Other works also found positive associations between air pollution and depressive and anxiety symptoms (Braithwaite et al., 2019; Zundel et al., 2022; Pun et al., 2017). Some authors have revised the literature and suggested that air pollution increases depressive and anxiety symptoms and behaviors, along with alterations in brain regions, leading to psychopathology risk (Zundel et al., 2022). The literature indicates that an increase in PM_2.5_ is significantly associated with anxiety symptoms, with the largest increase for the 180-day moving average in participants between 69 and 72 years old (Pun et al., 2017).

Neighborhood environmental characteristics play a relevant role in mental health during aging. Motoc et al. recently indicated that various neighborhood characteristics are associated with an increased incidence of anxiety, including higher urban density, higher PM_2.5_, and less green space (Motoc et al., 2023). Other studies have also found relationships between neighborhood characteristics and depressive or anxiety symptoms. For example, perceived social cohesion (Qin et al., 2023) and outdoor fitness facilities (Mu et al., 2023) are associated with a lower prevalence of depressive symptoms. These findings underscore the role of social characteristics in human health in the context of air pollution.

Social networks in the elderly may be sufficiently important to reduce the risk of mood disorders in older adults. In fact, Ward and colleagues found that greater social disconnection was associated with greater “wish to die,” with loneliness being the most important factor (Ward et al., 2024). Neighborhood is related to the services, accessibility, pollution, and social capital provided at the neighborhood level, and previous research has shown that it impacts both air quality and wellbeing (Lal et al., 2020). Similar to other emotional responses, Gu et al. (2020) suggested that exposure to a greater risk of air pollution might affect the mental health of the population by indirectly reducing wellbeing and social development. Thus, the concept of cognitive aging linked to brain toxicity secondary to environmental pollution in rural and urban areas does not explain the aging process in a global, unique, and direct way. A multidimensional vision is required that incorporates other factors associated with lifestyle, social support, and social networks in both areas.

## 7 Discussion and recommendations for future actions

Some studies have analyzed the role of residential settings and air pollution in the context of a behavioral frame in elderly people; however, they are insufficient. It seems that environmental agents could be the key to understanding the susceptibility and variability observed in behavioral symptoms during aging. The purpose of this review was to provide a deeper understanding of the cognitive, emotional, and social dimensions—both general and specific subdomains—which are influenced by air quality, place of residence, and individual susceptibility to environmental variations (Figure 2). Additionally, we highlight the role of social health and outline policy recommendations in the context of PM air pollution.

Aging is a universal phenomenon that does not necessarily have to bring the typical pattern of cognitive decline associated with dementia. Although we should note that cognitive decline is related to an increase in age, age is not a manipulated factor (Feldman and Kertesz, 2001; Kushwaha et al., 2017). Therefore, efforts should be directed to identify additional factors involved in the aging process, which could be susceptible to manipulation or variation. Might air quality be a key factor contributing to cognitive decline and mood disorders associated with aging?

### 7.1 PM air pollution associated with cognitive decline and mood disorders

Regarding behavioral effects, Table 1 shows the main effects of air pollution exposure on cognitive function in elderly people. The number of cases of dementia worldwide is increasing along with the number of air pollutants and urban areas. The repercussions of air pollution on the brain and central nervous system imply cognitive alterations. It has been reported that the exposure to air pollutants cause a decrease in the cognitive function leading to moderate to severe damage (Tallon et al., 2017; Gatto et al., 2014; Petkus et al., 2021; Ji et al., 2020; Wang et al., 2020; Lee et al., 2022; Power et al., 2011; Cho et al., 2023; Gui et al., 2024; Lo et al., 2019; Tallon et al., 2017; Gatto et al., 2014; Petkus et al., 2021; Ji et al., 2020; Wang et al., 2020; Lee et al., 2022; Power et al., 2011; Cho et al., 2023; Gui et al., 2024; Lo et al., 2019; Tallon et al., 2017; Gatto et al., 2014; Petkus et al., 2021; Ji et al., 2020; Wang et al., 2020; Lee et al., 2022; Power et al., 2011; Cho et al., 2023; Gui et al., 2024; Lo et al., 2019; Tallon et al., 2017; Gatto et al., 2014; Petkus et al., 2021; Ji et al., 2020; Wang et al., 2020; Lee et al., 2022; Power et al., 2011; Cho et al., 2023; Gui et al., 2024; Lo et al., 2019). Several studies have observed notable cognitive declines associated with long-term exposure to urban pollution levels (Weuve et al., 2012; Zeng et al., 2010; Power et al., 2011). Some researchers have even reported a 2-year accelerated decline in cognitive function for every 10 mg/m^3^ increase in long-term PM exposure among older women (Weuve et al., 2012). Air pollution has also been linked to neurodegenerative disorders (Dimakakou et al., 2018; Costa et al., 2020). Specifically, the authors mark poorer performance in executive functions, lower olfactory function, deficits in working memory, episodic memory, verbal learning, attentional processes, impulsivity behavior and higher levels of mortality as the mains cognitive alterations (Ranft et al., 2009; Gatto et al., 2014; Siddique et al., 2011; Young and Bok, 2017; Ailshire and Clarke, 2015; Ailshire and Crimmins, 2014; Rivas et al., 2019; Sacks et al., 2011). On top of that, it seems that exist a higher incidence of dementia in cities with poor air quality (Costa et al., 2020; Braithwaite et al., 2019; Chang et al., 2014; Jung et al., 2015; Wu et al., 2015; Chen et al., 2016; Kioumourtzoglou et al., 2016). The authors specifically inform of higher risk of AD, PD and VD among the neurodegenerative diseases (Parra et al., 2022; Costa et al., 2020; Shim et al., 2023).

In addition, air quality is implicated in a range of emotional symptoms such as depression, anxiety and emotional wellbeing responses (Power et al., 2015; Lim et al., 2012; Sass et al., 2017; Altuğ et al., 2020; Barnett et al., 2018; Gu et al., 2020; Pun et al., 2017; Lu, 2020; Li and Zhou, 2020) (Table 2). Recent research has shown a positive association between air pollutants and depressive symptoms, even with a higher risk of suicidal behavior (Young et al., 2018). Moreover, older women seem to be more susceptible to developing depressive symptoms under air pollution conditions (Altuğ et al., 2020). Likewise, subjective environmental evaluations can modulate the link between objective environmental quality and life satisfaction by measuring feelings of wellbeing feeling (Li and Zhou, 2020; Liao et al., 2015). In general, these studies show a negative association between air pollution, life satisfaction, and happiness, indicating a negative impact of air pollution on emotional human responses. Some of these studies have shown that higher levels of air pollution, as well as living within 100 m of a major road, were significantly positively associated with emotional symptoms, foregrounding the relevance of residential settings (Altuğ et al., 2020).

### 7.2 Increased susceptibility to environmental pollution in older adults

Sacks and colleagues established older adults as a vulnerable group to health effects related to PM exposure (Sacks et al., 2011). In addition, this research group was able to develop a definition of susceptibility that could be applied to include all populations potentially at risk of negative health effects due to air pollutant exposure. Aging is a natural process characterized by the gradual, time-dependent decline in biological and behavioral functions, with individuals of the same chronological age exhibiting variability (Sacks et al., 2011; Martins-De-Souza, 2023). Older adults are considered a susceptible population due to this gradual decline. Furthermore, older adults are considered more susceptible than younger groups because of the higher prevalence of preexisting cardiovascular and respiratory conditions, which may further increase their vulnerability to PM exposure (Sacks et al., 2011). With advancing age, studies on the dosimetry model of PM—which examines how these particles are deposited, accumulated, and cleared in different regions of the respiratory tract—have shown a reduced clearance of PM across all regions of the respiratory system (EPA, 2009).

So that, PM enters the respiratory tract via the nose to the olfactory bulb and travel into the brain, making it especially vulnerable (López-Granero et al., 2024; Costa et al., 2019; Costa et al., 2021). In fact, impaired olfactory function has been proposed as a precursor of AD (Calderon-Garciduenas et al., 1995; Fagundes et al., 2015). In this sense, some evidence also suggests an increased accumulation of amyloid-β in older individuals exposed to elevated levels of air pollution (Calderón-Garcidueñas et al., 2004). Therefore, PM pollutants may disrupt the blood-brain barrier and deposit in neural tissue, leading to systemic inflammation (Block et al., 2012). The inflammatory and oxidative stress responses triggered by air pollution are similar to those observed during natural aging, and the effects of these systems have been reported in the hypothesized biological pathways of neurological disorders (Dröge and Schipper, 2007; Schikowski and Altuğ, 2020).

Air pollution is not only associated with a higher susceptibility to respiratory and cerebrovascular systems but is also associated with CVD (Clifford et al., 2016; Zhou et al., 2024). Precisely, these systems show a decline with advancing age; therefore, environmental exposure could contribute to further deterioration. It has been reported increased cardiovascular disease hospital admissions among older adults compared with all ages when exposed to PM_2.5_ (Sacks et al., 2011; Pope et al., 2008). In this study, exacerbation of heart failure was associated with elderly patients, and the authors concluded that PM air pollution could be responsible for precipitating acute cardiac decompensation in otherwise well-managed patients with heart failure (Pope et al., 2008). A recent study observed that increasing exposure to PM_2.5,_ was significantly associated with an increased risk of gastrointestinal bleeding, intracranial bleeding, and epistaxis in older adults (Fayyad et al., 2024).

### 7.3 The role of social health in mediating the effects of air pollution

The role of social health in pollution-free residences in elderly people deserves to be discussed. Some authors (Blekesaune and Haugen, 2018) offered a specific view of the importance of treating the linkage between the elderly and the community to obtain a better quality of life. Many older adults living in rural municipalities reported a stronger sense of community than those living in urban municipalities (Verheij et al., 2008). Maintaining positive and lasting social relationships with friends and neighbors is essential for the quality of life of the elderly (Bowling, 2005; Scharf, 2001), and the neighborhood is a particularly important source of social contact for the elderly (Blekesaune and Haugen, 2018). Perceptions of who is considered a neighbor can vary between urban areas (less knowledge) and rural areas (greater knowledge). Performing and receiving informal services in a locality is a good indicator of mutual relationships and support. Older rural residents have closer contact with their neighbors than urban dwellers. Friendships and neighborhoods tend to overlap more in rural communities than in urban areas. A higher proportion of those living in rural areas rely on getting help from friends in the community, suggesting that local friendship networks are stronger in rural municipalities than in urban ones (Blekesaune and Haugen, 2018).

Regarding social exclusion in old age, it seems that older people living in urban neighborhoods have higher levels of social exclusion. Biopsychosocial community deprivation has a high impact on the neighborhood and directly affects a greater proportion of older people. In this sense, it is explained that people who live in urban areas have a greater risk of social exclusion due to their predisposition to fewer ties with the neighborhood, due to the greater probability of deprivation and the loss of economic status in urban areas. Residents’ connections to their neighborhoods have the power to mitigate exclusion (Barnes et al., 2006; Jivraj et al., 2016).

We also conclude that the level of social networks and support inherent to a rural area in the elderly may be sufficiently important to reduce the risk of at least being seen at the emotional stage when access to essential services is guaranteed. Air contamination can modulate cognitive and emotional variables during aging, having strong and adequate social relationships with friends and neighbors is an essential value in the elderly for the quality of life (Bowling, 2005; Scharf, 2001) and this kind of connecting links are more suitable in rural areas. The perception of whoever is considered a neighbor is stronger in rural areas (Braithwaite et al., 2019; Zundel et al., 2022; Pun et al., 2017). It seems that older people living in rural areas obtain higher scores in self-perception of health, and this is attributed to the influence of the rural context on health (perception of natural environments as healthier, being away from pollution, and stress that experiences living in more industrialized urban areas) (Benavente Cuesta and Quevedo Aguadode, 2019) and the highest levels of exclusion found in urban populations compared to rural ones (Walsh et al., 2017).

### 7.4 Implications for society and policy recommendations

This work offers a comprehensive analysis of the impact of air quality on human health during aging. Identifying vulnerable population groups, such as older adults, affected by air pollution will aid in the development of effective prevention programs and the strategic planning of targeted interventions for specific populations. The health impacts of ambient air pollution result in significant costs to society (Yin et al., 2021). Higher health costs are associated with older people than with younger people. According to Yin and collaborators, in 2016, PM_2.5_ was estimated to have caused 8.42 million attributable deaths, which was associated with 163.68 million years of life lost (Yin et al., 2021). The economic cost *per capita* for the older population was $2,739, which was 10 times that of the younger population.

Consequently, policy strategies should focus on reducing the health effects and costs of ambient air pollution, with particular emphasis on the older population. Since 2005, the WHO has been implementing Global Air Quality Guideline recommendations, with the latest update published in 2022. Recent research suggests that efforts to address air pollution require both local and regional interventions, as well as global cooperation (Kandpal, 2024; Ogwu et al., 2024). Some of the proposed measures include the implementation of pollution prevention and control measures in industries that could help minimize the emissions of pollutants such as sulfur dioxide, nitrogen oxides, PM, and volatile organic compounds (McDonald et al., 2020). At the governmental level, national authorities are encouraged to monitor emissions, track air quality levels, and apply corrective measures to address non-compliance with regulatory standards. At the individual level, masks have consistently been identified as effective tools against environmental threats (Tcharkhtchi et al., 2021). They are considered to be protective equipment designed to protect the respiratory system from airborne droplets, viral particles, and pollutants. However, the filtration efficiency of various masks against aerosols is not uniform because the particles differ in size, shape, and properties. This underscores the necessity of using specific masks to ensure optimal protection (Tcharkhtchi et al., 2021).

**FIGURE 6:**
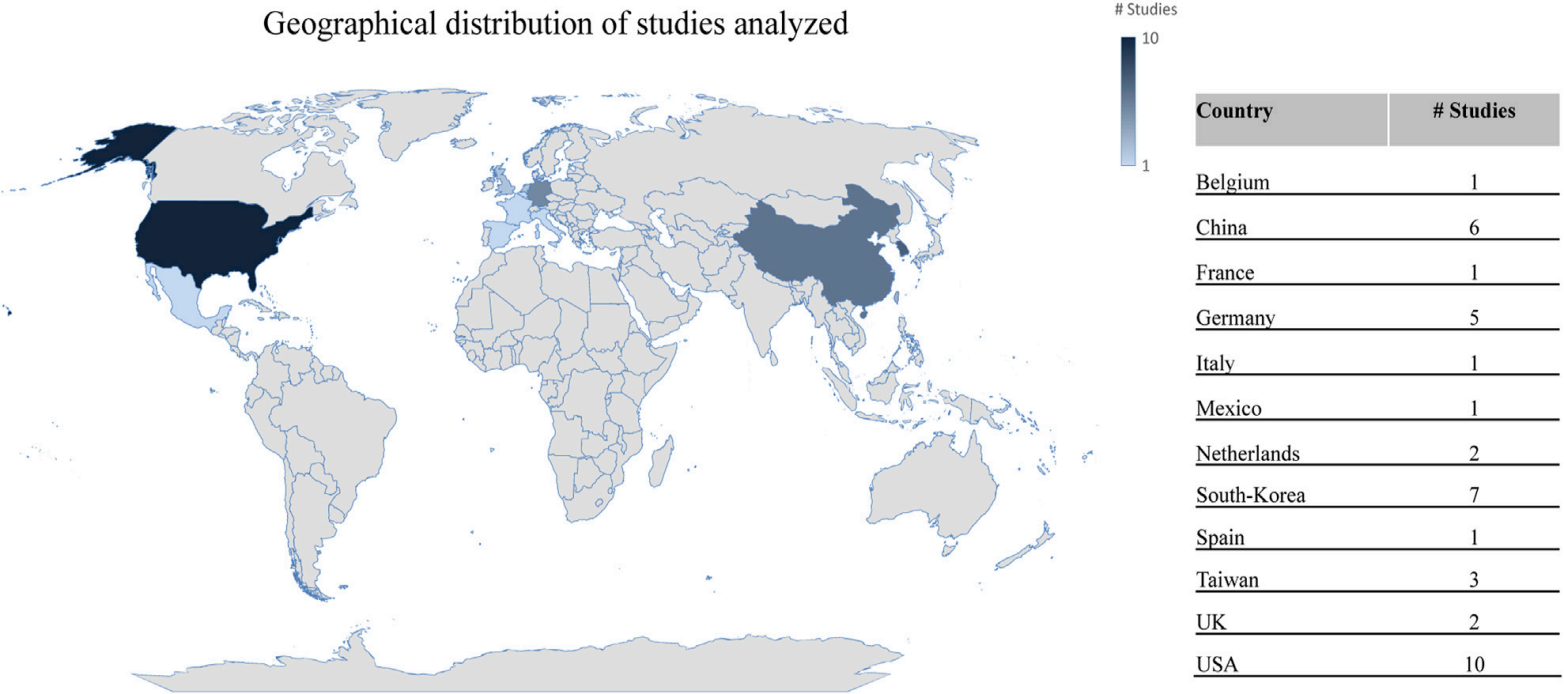
Geographical distribution of studies analyzed in relation to cognitive function, mood disorders, and PM air pollution.

Figure 6 illustrates the geographical distribution of the studies analyzed in this study. It is worth noting that these countries appear to undertake governmental actions against air pollution (Schraufnagel et al., 2019). Most developed nations have high-tech equipment and monitoring systems to collect and analyze air quality data, which are then used to generate information that informs policies and the population at local, national, and regional levels (United Nations Environment, 2019). However, in underdeveloped countries, such as those on the African continent, access to this technology remains limited (United Nations Environment, 2019). Promoting air quality policies and developing studies, such as those mentioned above, can help reduce the risk of exposure to air pollution and, consequently, the incidence of certain pathologies in humans. In 2015, Adar et al. studied the impact of clean-air technologies and fuel policies in Washington (US). The transition to low-sulfur diesel was associated with improved lung function (Adar et al., 2015). Similarly, in Korea, associations have been found between a decrease in pollutant emissions and hospital visits for asthma (Hu et al., 2022; Chen et al., 2020). In 2013, China launched a plan known as the Air Pollution Prevention and Control Action Plan (AAPCAP), which aimed to improve air quality through actions such as optimizing industrial structures, developing clean energy, establishing early warning and emergency monitoring systems, and reducing the use of dirty fuels (Hu et al., 2022; Chen et al., 2020), primarily focusing on the reduction of PM. As a result, within just 4 years (by 2017), there was a 33.3% reduction in the annual average concentration of PM_2.5_ compared to 2013, when the project commenced (Hu et al., 2022). This reduction also led to improvements in certain health indicators, such as a lesser decline in cognitive function (Younan et al., 2022; Hu et al., 2022; Yao et al., 2022b). Schraufnagel et al. analyzed the benefits of air pollution reduction in the US, Western Europe and Asia (Schraufnagel et al., 2019).

## 8 Conclusion

Finally, we posit that it is necessary to apply a multidimensional approach that incorporates cognitive, emotional, and social elements to understand the human aging model. Based on the analyzed literature, we conclude that (a) air pollutants affect cognitive function and increase the risk of dementia and neurodegenerative disorders; (b) there is a link between depression, anxiety, and wellbeing emotional responses and poor air quality and risk of suicide; (c) social networks and inclusion can modulate and mitigate the effects observed during aging in rural areas exposed to less contamination; (d) older adults are particularly susceptible and vulnerable to the effects of PM pollution; (e) the consequences of air pollution exposure are not specific to a given country; and (f) new human epidemiological studies are necessary to better explain individual differences during aging in rural/urban residential settings. It is well known that pollution is caused by anthropogenic secondary to rapid industrialization and urbanization, accordingly, it is time to reverse this tragic trend by means of law that reduces air contamination. Pollution must be addressed at its source, emphasizing the need for increased efforts to reduce pollution at several levels, including global, local, and personal-level interventions.
